# Clinicopathological features of adult T‐cell leukemia/lymphoma with T‐follicular helper phenotype

**DOI:** 10.1002/cam4.7050

**Published:** 2024-03-20

**Authors:** Reiji Muto, Hiroaki Miyoshi, Kazutaka Nakashima, Mai Takeuchi, Makoto Hamasaki, Koichi Ohshima

**Affiliations:** ^1^ Department of Pathology, National Hospital Organization (NHO) Kumamoto Medical Center Kumamoto Japan; ^2^ Department of Pathology Kurume University School of Medicine Kurume Japan; ^3^ Department of Pathology Fukuoka University School of Medicine Fukuoka Japan

**Keywords:** adult T‐cell leukemia/lymphoma, malignant lymphoma, T‐cell lymphoma, T‐follicular helper phenotype

## Abstract

**Aims:**

T‐follicular helper (TFH) cells are effector T‐cells that are crucial for B‐cell selection and differentiation. T‐cell lymphomas derived from TFH cells have distinct characteristics. Additionally, in the World Health Organization (WHO) classification 5th edition, three lymphomas were introduced as independent disease entities with TFH cell origin. We aimed to investigate the clinicopathological features of adult T‐cell leukemia/lymphoma (ATLL) with a TFH phenotype (TFHP).

**Methods and Results:**

We performed TFH immunohistochemistry analysis of five biomarkers for the biopsy specimen, with TFHP being indicated by a positive result for more than two markers. Among 75 cases of ATLL, 37.3% of them showed TFHP. Compared with cases of ATLL without TFHP, cases of ATLL with TFHP showed higher C‐reactive protein levels (*p* = 0.0219) and increased high endothelial venule proliferation (*p* = 0.024). However, there were no significant between‐group differences in overall survival as well as other clinical and morphological findings. Furthermore, there was no significant between‐group difference in TFH markers and FOXP3 expression.

**Conclusion:**

Some patients with ATLL may present a TFHP, which should not preclude the diagnosis of ATLL. Although presenting a TFHP does not affect prognosis, it is important to identify cases of ATLL with a TFHP since it may inform future treatment strategies.

## Graphical abstract

Adult T‐cell leukemia/lymphoma (ATLL) may present with T‐follicular helper (TFH) markers, and ATLL with TFH phenotype present high endothelial venule proliferation and higher C‐reactive protein levels than non‐TFH phenotype but does not affect the prognosis. It may be important to stratify treatment interventions for ATLL according to the presentation of THFP.

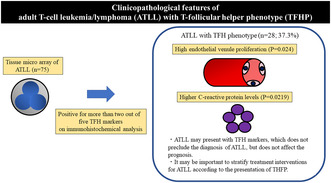

## 1 INTRODUCTION

Adult T‐cell leukemia/lymphoma (ATLL) is a subtype of peripheral T‐cell lymphoma (PTCL) that is caused by the human retrovirus known as human T‐cell leukemia virus type 1 (HTLV‐1).^1^ Based on the Shimoyama classification, there are four clinical variants of ATLL, including the acute variant (characterized by leukemic phase), lymphomatous variant (characterized by lymphadenopathy), chronic variant, and smoldering variant.^2^ The former two variants have a poor prognosis,^2^, ^3^ with 4‐year survival rates of 16.8% and 19.6%, respectively.^2^ Although the latter two variants have a relatively good prognosis, approximately 25% of the chronic and smoldering variants transform into leukemia after a long duration.^2^, ^3^

T follicular helper (TFH) cells are a subset of effector T‐cells that are crucially involved in B‐cell selection and differentiation to plasma cells and memory B‐cells. Furthermore, these cells express anti‐programmed cell death protein 1 (PD‐1), inducible T‐cell co‐stimulator (ICOS), CD10, B‐cell lymphoma (BCL) 6, chemokine (C‐X‐C motif) ligand 13 (CXCL13), and CD4.^4^ The 2017 WHO classification introduced a new independent subtype of PTCL termed nodal PTCL with TFH phenotype (nPTCL with TFHP), and this category is essentially followed by the WHO 5th edition: nodal TFH cell lymphoma, not otherwise specified (nTFHL‐NOS).^1^ This subtype is characterized by lymphoma cells with a TFHP; however, it is difficult to morphologically distinguish it from PTCL and NOS. Genetic studies have reported mutations in nTFHL‐NOS, including in Ten‐Eleven Translocation 2, Ras homolog family member A (RHOA), and DNA‐methyltransferase 3 alpha.^1^ Although the role of RHOA remains unknown, its expression in CD4 + T cells induces TFH cell specification, including increased proliferation related to ICOS upregulation.^5^ RHOA mutations have also been found in 15% of ATLL cases.^6^ The histological characteristics of ATLL range from small atypical lymphocytes to large ones; accordingly, there remain no established findings for ATLL.^7^ Therefore, measurement of serum HTLV‐1 antibody levels is important in the diagnosis of ATLL. In clinical practice, serum HTLV‐1 antibodies are often not examined during histological examination. Therefore, pathologists may encounter cases that were initially diagnosed as nTFHL‐NOS but were later shown to be ATLL. However, the characteristics of ATLL with TFHP remain unclear.

We aimed to review the clinicopathological characteristics of ATLL with TFHP.

## 2 MATERIALS AND METHODS

### 2.1 Tissue samples

We studied 75 patients diagnosed with ATLL between 2006 and 2012 at the Department of Pathology, Kurume University. We used a tissue microarray that has been analyzed in previous studies.^8^, ^9^, ^10^ We excluded cases of recurrence. This study was approved by the Research Ethics Committee of Kurume University and conducted following the Declaration of Helsinki.

### 2.2 Morphology and immunohistochemistry

We morphologically examined the paraffin sections of each sample. Lymphoma cells were identified based on their morphological characteristics, including size and variation of the atypical lymphocyte, nucleus, and nucleoli, nuclear shape irregularity, and hyperchromatism with a coarse and irregular distribution. Additionally, morphological variants, including small‐, medium‐, large‐, and anaplastic‐cell variants; pleomorphic variants; resembling angioimmunoblastic T‐cell lymphoma (AITL: In the WHO 5th edition, the name was changed to nTFHL‐angioimmunoblastic type (AI), but the definition is different from AITL, so the conventional name AITL is used in the part that introduces previous research.) variants; and Hodgkin‐like variants, were classified based on previously reported histological features.^8^, ^11^, ^12^ For each specimen, the number of eosinophils and high endothelial venules (HEVs) was evaluated at high magnification in three fields at hot spots.

**FIGURE 1:**
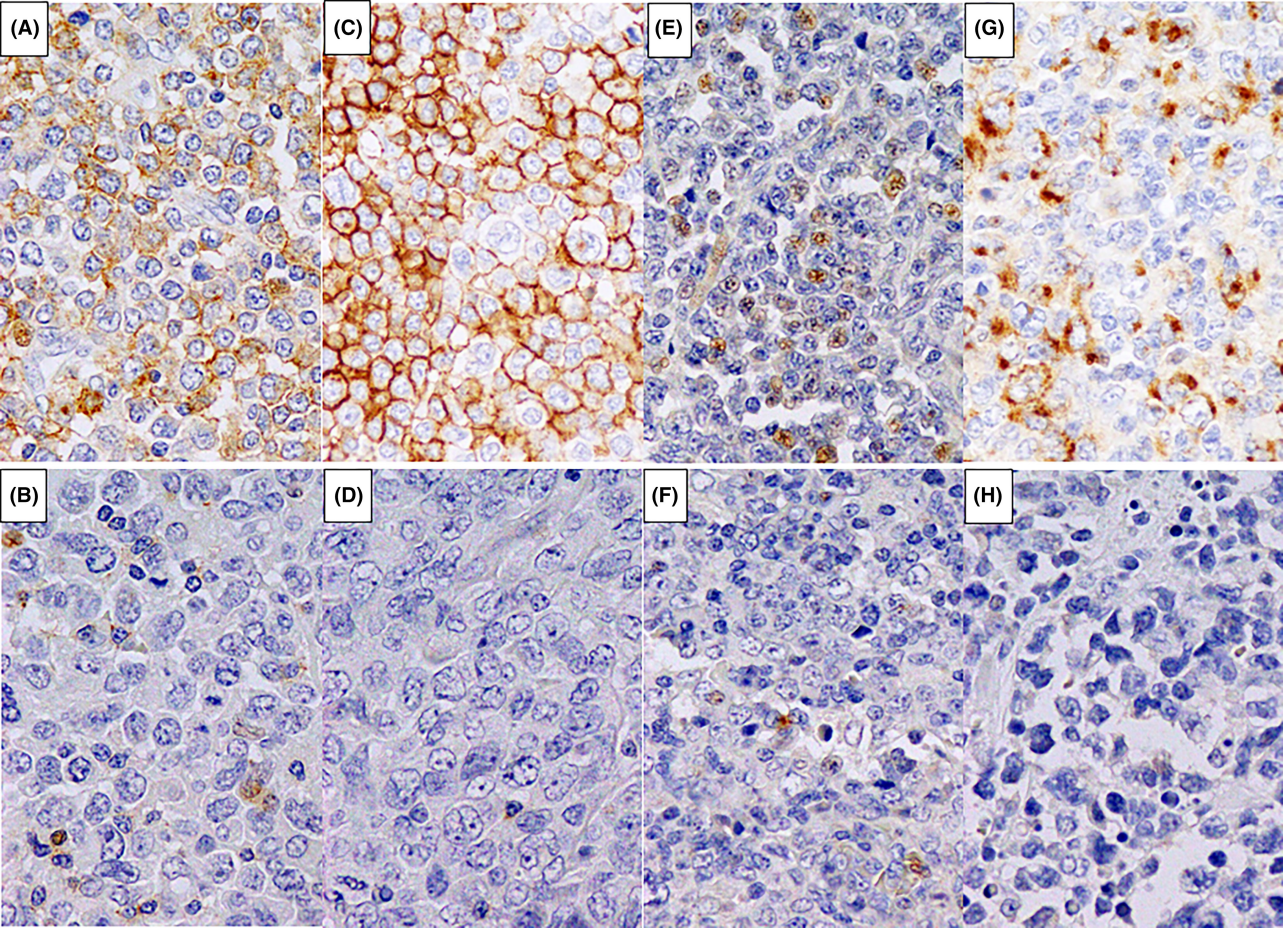
Representative immunohistochemical analysis in adult T‐cell lymphoma/leukemia. The upper row presents positive cases and the lower row presents negative cases in each follicular helper marker; PD‐1 (A, B), ICOS (C, D), BCL6 (E, F), and CXCL13 (G, H). Original magnification 400 for all panels.

We performed immunohistochemistry analyses of formalin‐fixed, paraffin‐embedded tissues. All sections underwent hematoxylin and eosin staining and were immunostained for CD3 (F7.2.38; DAKO JAPAN, Kyoto, Japan), CD4 (4B12; Leica Biosystems, Tokyo, Japan), PD‐1 (NAT105; Abcam, Tokyo, Japan), ICOS (D1K2T; Cell Signaling Technology, Danvers, MA, USA), CD10 (56C6; Nichirei Biosciences Inc., Tokyo, Japan), BCL6 (PG‐B6p; DAKO JAPAN, Kyoto, Japan), and CXCL13 (53,610; R&D Systems, Minneapolis, MN, USA). Two experienced hematopathologists (R.M. and H.M.) examined the immunohistochemical results. Specimens were considered positive for a marker if >30% of the lymphoma cells were immunoreactive for the antibody. Cases that were positive for more than two of the aforementioned TFH markers were considered ATLL with TFHP. Figure 1 shows a representative immunohistochemical analysis. Regarding the positivity for forkhead/winged helix transcription factor (FOXP3) (SP97; Abcam), we used data obtained in a previous study.^8^

### 2.3 Statistical analysis

Statistical analyses were conducted using JMP Pro 16 software. Statistical significance was set at *p* values < 0.05. Overall survival (OS) was defined as the duration from the date of diagnosis to the date of death or last follow‐up. The Kaplan–Meier method was used to estimate the distribution of OS, which was analyzed using the log‐rank test. Between‐group comparisons of clinicopathological characteristics were performed using the Chi‐square test, Fisher's two‐sided exact test, or the Mann–Whitney *U* test.

## 3 RESULTS

### 3.1 Immunohistochemical results

Among 75 ATLL cases, 7 (9.3%) and 21 (28.0%) cases were positive for three and two TFH marker, respectively; accordingly, 28 (37.3%) cases were assigned as ATLL with TFHP. None of the cases were positive for four or five TFH markers. The distribution of identified TFHP markers was as follows: PD‐1, 20 (26.7%) cases; ICOS, 51 (68.0%) cases; CXCL13, 8 (10.7%) cases; BCL6, 10 (13.3%) cases; and CD10, 0 (0%) cases. Among three‐marker positive cases, PD‐1, ICOS, and BCL6 were the most common (4/7 cases: 57.1%), followed by the combination of ICOS, CXCL13, and BCL6 in 2 cases (28.6%) and PD‐1, ICOS, and CXCL13 in 1 case (14.3%). Among the two‐marker positive cases, the combination of PD‐1 and ICOS was the most common (14/21 cases: 66.7%), followed by ICOS and CXCL13 in 5 cases (23.8%) and ICOS and BCL6 in 2 cases (9.5%).

### 3.2 Clinical characteristics of ATLL with TFHP

Table 1 shows the patient characteristics. Only elevated C‐reactive protein (CRP) levels (*n* = 70, 26 TFHP cases and 44 non‐TFHP cases, *p* = 0.0219) showed a significant difference. Other clinical findings were as follows: B‐symptoms (*n* = 66, 23 TFHP cases and 43 non‐TFHP cases, *p* = 1.0000), skin lesion (*n* = 70, 26 TFH cases and 44 non‐TFHP cases, *p* = 0.1880), splenomegaly (*n* = 70, 26 TFHP cases and 44 non‐TFHP cases, *p* = 0.2270), hepatomegaly (*n* = 70, 26 TFHP cases and 44 non‐TFHP cases, *p* = 1.0000), pleural effusion / ascites (*n* = 70, 26 TFHP and 44 non‐TFHP cases, *p* = 1.0000), hemolytic anemia (*n* = 66, 24 TFHP cases and 42 non‐TFHP cases, *p* = 1.0000), presence of autoantibody (*n* = 16, 6 TFHP cases and 10 non‐TFHP cases, *p* = 1.0000), clinical subtypes (acute / lymphoma type; *n* = 46, 17 TFHP cases and 29 non‐TFHP cases, *p* = 0.7614), elevated lactate dehydrogenase levels (*n* = 71, 27 TFHP cases and 44 non‐TFHP cases, *p* = 0.5516), hypergammaglobulinemia (*n* = 48, 16 TFHP cases and 32 non‐TFHP cases, *p* = 1.0000), hypercalcemia (*n* = 71, 27 TFHP cases and 44 non‐TFHP cases, *p* = 0.896), elevated beta 2 micro‐globulin levels (*n* = 17, 6 TFHP cases and 11 non‐TFH cases, *p* = 1.0000), performance status 0–1 or 2–4 (*n* = 68, 25 TFHP cases and 43 non‐TFH cases, *p* = 0.7978), extra‐nodal site involvement (*n* = 67, 26 TFH cases and 41 non‐TFP cases, *p* = 0.6039), peripheral blood involvement (*n* = 69, 25 TFH cases and 44 non‐TFH cases, *p* = 0.4613), bone marrow involvement (*n* = 43, 17 TFH cases and 26 non‐TFH cases, *p* = 0.3443), Ann arbor stage I–II or III–IV (*n* = 70, 27 TFHP cases and 43 non‐TFHP cases, *p* = 1.0000), high or low Japan Oncology Group‐prognostic index (*n* = 68, 25 TFHP cases and 43 non‐TFHP cases, *p* = 0.7115), and complete remission (CR) (u) or non‐CR(u) (*n* = 57, 20 TFHP cases and 37 non‐TFHP cases, *p* = 0.1278).

**TABLE 1:** Clinical characteristics of ATLL patients in the present study.

Clinical features	Total	%	TFHP	%	non‐TFHP	%	*p*‐Value
Age median (range)	70 (13–90)	–	67.8 (47–86)	–	67.4 (13–90)	–	–
Sex female/male	34/41	–	15/13	–	19/28	–	–
Shimoyama classification							
Smoldering	2/48	4.2	2/19	11.8	0/29	0.0	–
Acute	19/48	39.6	8/19	42.1	11/29	37.9	–
Lymphoma	27/48	56.3	9/19	47.4	18/29	62.1	–
B symptoms	18/66	27.3	6/23	26.1	12/43	27.9	1.0000
Skin lesion	12/70	17.1	2/26	7.7	10/44	22.7	0.1880
Hepatomegaly	6/70	8.6	2/26	7.7	4/44	9.1	1.0000
Splenomegaly	15/70	21.4	8/26	30.8	7/44	15.9	0.2270
Pleural effusion/Ascites	5/70	7.1	2/26	7.7	3/44	6.8	1.0000
Hemolytic anemia	1/66	1.5	1/24	4.2	0/42	0.0	1.0000
Autoantibody	2/16	12.5	1/6	16.7	1/10	10.0	1.0000
Performance status, 2–4	27/68	39.7	9/25	36.0	18/43	41.9	0.7978
Extra‐nodal site involvement	43/67	64.2	18/26	69.2	25/41	61.0	0.6039
Bone marrow involvement	18/43	41.9	9/17	52.9	9/26	34.6	0.3443
Peripheral blood involvement	37/69	53.6	15/25	60.0	22/44	50.0	0.4613
Ann Arbor stage, III–IV	57/70	81.4	22/27	81.5	35/43	81.4	1.0000
Elevated LDH	56/71	78.9	20/27	74.1	36/44	81.8	0.5516
Elevated β2‐microglobulin	13/17	76.5	6/6	100.0	7/11	63.6	1.0000
Hypergammaglobulinemia	7/48	14.6	2/16	12.5	5/32	15.6	1.0000
Hypercalcemia	11/71	15.5	7/27	25.9	4/44	9.1	0.8960
Elevated CRP	44/70	62.9	21/26	80.8	23/44	52.3	0.0219
JCOG‐PI, high	31/68	45.6	12/25	48.0	19/43	44.2	0.7115
IPI, high‐intermediate or more	49/67	73.1	20/26	76.9	29/41	70.7	0.7781
Treatment							
Chemotherapy	59/70	84.3	22/27	81.5	37/43	86.0	1.0000
Radiation	2/68	2.9	1/25	4.0	1/43	2.3	0.1699
Transplantation	10/67	14.5	3/25	12.0	7/42	16.7	0.2787
CR or CR(u)	17/57	29.8	3/20	15.0	14/37	37.8	0.1278

Abbreviations: CR(u), complete response/remission or uncertain complete response/remission; CRP, C‐reactive protein; IPI, international prognostic index; JCOG‐PI, Japan clinical oncology group‐prognostic index; LDH, lactate dehydrogenase; TFHP, T‐follicular helper phenotype.

### 3.3 OS in ATLL with TFHP

**FIGURE 2:**
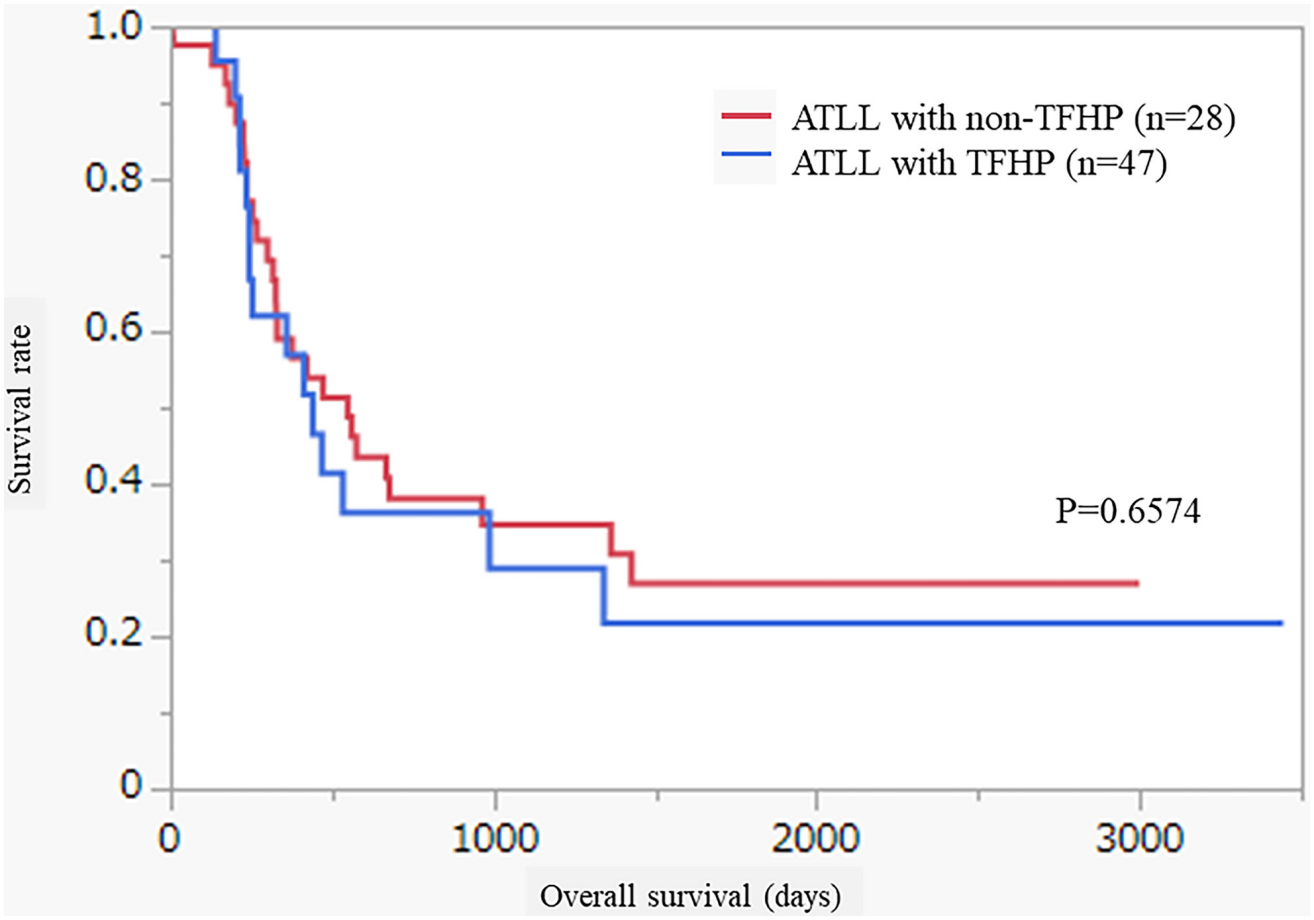
Overall survival (OS) in adult T‐cell lymphoma/leukemia with T‐follicular helper phenotype (TFHP). OS was compared between TFHP cases and non‐TFHP cases, but no significant difference was observed (*p* = 0.6574).

There was no significant difference in OS between cases of ATLL with TFHP (median survival time: 249 days, mean survival days: 582.8 days) and without TFHP (median survival time: 399.5 days, mean survival days: 703.2 days; *p* = 0.6574; Figure 2).

### 3.4 Pathological characteristics of ATLL with TFHP

We performed between‐group comparisons of pathological characteristics (Tables 2 and 3). Among cases of ATLL with TFHP, there were five (17.9%) cases of medium‐cell variants; 15 (53.6%) cases of large‐cell variants; one (3.6%) case of the anaplastic variant; seven (25.0%) cases of pleomorphic variants; and no cases of the small‐cell, resembling AITL, and Hodgkin‐like variants. Contrastingly, cases of ATLL without THPF included one (2.1%) case of the small‐cell variant, 10 (21.3%) cases of the medium‐cell variant, 18 (38.3%) cases of the large‐cell variant, two (4.3%) cases of the anaplastic variant, 15 (31.9%) cases of the pleomorphic variant, one (2.1%) case of the Hodgkin‐like variant, and no case of the resembling AITL variant. In the stromal cells, there was a significant difference in the number of HEVs (*p* = 0.024: TFHP group:4.3/3.7 [0.7–9.7] and non‐TFHP group:3.3/2.5 [0–12.7] (average/median [range])), but not eosinophils (*p* = 0.724), in tumor tissue.

**TABLE 2:** Pathological characteristics of ATLL: morphological variants of lymphoma cells.

Morphological Variant	All cases (*n* = 75)	ATLL with TFHP (*n* = 28)	ATLL with non‐TFHP (*n* = 47)
Small cell variant	1 (1.3%)	0 (0%)	1 (2.1%)
Medium cell variant	15 (20%)	5 (17.9%)	10 (21.3%)
Large cell variant	33 (44%)	15 (53.6%)	18 (38.3%)
Anaplastic variant	3 (4%)	1 (3.6%)	2 (4.3%)
Pleomophic variant	22 (29.3%)	7 (25%)	15 (31.9%)
Resembling AITL	0 (0%)	0 (0%)	0 (0%)
Hodgkin like variant	1 (1.3%)	0 (0%)	1 (2.1%)

Abbreviations: AITL, angioimmunoblastic T‐cell lymphoma; ATLL, adult T‐cell lymphoma/leukemia; TFHP, T‐follicular helper phenotype.

**TABLE 3:** Pathological characteristics of ATLL: morphological evaluation of stromal cells.

	All cases (*n* = 74)	ATLL with TFHP(*n* = 28)	ATLL with non‐TFHP(*n* = 46)	*p*‐Value
Infiltration of eosinophils, average/median[range] (counts/HPF)	9.2/0.3[0–177]	3.4/0.3[0–65.7]	12.7/0.3[0–177]	0.724
Proliferation of HEV, average/median[range] (counts/HPF)	3.7/3.3[0–12.7]	4.3/3.7[0.7–9.7]	3.3/2.5[0–12.7]	0.024

*Note*: One case of ATLL with non‐TFHP was excluded because there were few evaluable areas in the specimen.

Abbreviations: HEV, high endothelial venule; HPF, high power field.

### 3.5 Number of positive T‐follicular helper markers and expression of FOXP3

**FIGURE 3:**
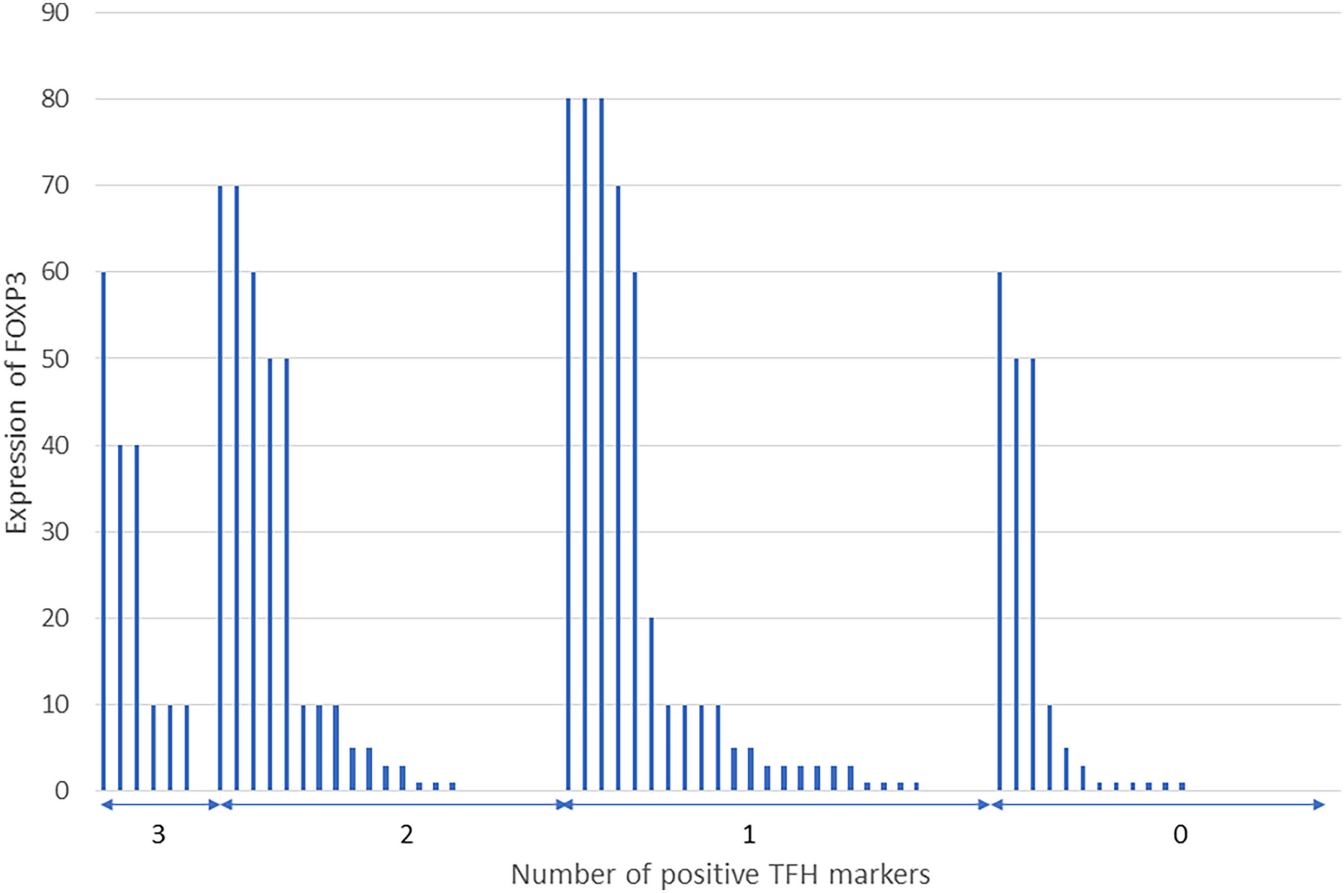
The relationship between the number of T‐follicular helper (TFH) markers and their expression rates in FOXP3 in lymphoma cells. The vertical axis shows the percentage of FOXP3‐positive cells among the lymphoma cells, while the horizontal axis shows the number of positive TFH markers. There was no relationship between the expression of TFH markers and FOXP3.

Figure 3 shows the number of positive TFH markers and their FOXP3 expression rates in lymphoma cells. The vertical axis shows the percentage of FOXP3‐positive cells among the lymphoma cells, while the horizontal axis shows the number of positive TFH markers. There was no relationship between the expression of TFH markers and FOXP3.

## 4 DISCUSSION

T‐follicular helper cells are a subset of T‐cells whose function has been examined since the early 2000s. In non‐neoplastic lymph nodes, TFH cells are localized in the germinal center of the lymph follicle and are crucially involved in selecting B‐cells, inducing antibody production, directing B‐cell differentiation, and class switching of B‐cells.^4^ Since the discovery of TFH cells, there have been reported cases of PTCLs with phenotypic features of TFH. nTFHL‐AI, nTFHL‐follicular type, and nTFHL‐NOS have been listed as lymphomas with a TFH cell origin according to the WHO classification of tumors of hematopoietic and lymphoid tissue, 5th edition.^1^ Although the precise role of RHOA remains unknown, expression of RHOA in CD4 + T cells induces TFH cell specification including increased proliferation related to ICOS upregulation.^5^

Adult T‐cell leukemia/lymphoma is generally considered to have a poor prognosis, and it has widely varying histological characteristics. There are cases that are composed of small‐sized atypical lymphocytes to composed of large‐sized ones. Additionally, some cases of ATLL are similar to the AITL, anaplastic large cell lymphoma, and Hodgkin lymphoma. The diagnostic clue for ATLL is confirming the positivity of serum HTLV‐1 antibody.^1^, ^7^, ^11^, ^12^ Recently, it has been reported that some ATLL cases are positive for TFH markers, but no study has examined their comprehensive clinical characteristics.^13^ In this study, in addition to the pathological analysis of ATLL with TFHP, clinical characteristics and prognosis were also examined.

In our study, among 75 ATLL cases, 7 (9.3%) and 21 (28.0%) cases were positive for three and two markers, respectively; accordingly, as many as 28 (37.3%) cases were assigned as ATLL with TFHP based on the WHO classification concept for TFHP. Regarding immunohistochemistry, PD‐1 and ICOS are generally considered the most sensitive markers, while CXCL13 and CD10 are considered the most specific markers for TFHP.^1^ In our study, the distribution of identified TFHP markers was as follows: PD‐1, 20 (26.7%) cases; ICOS, 51 (68.0%) cases; CXCL13, 8 (10.7%) cases; BCL6, 10 (13.3%) cases; and CD10, 0 (0%) cases.

In both groups, the most common morphological variant was the large‐cell variant, followed by the pleomorphic and medium‐cell variants. These three variants accounted for 96.5% and 91.5% of cases of ATLL with and without TFHP, respectively. Accordingly, we observed no between‐group difference in the tumor morphology.

Regarding stromal cells in AITL, which is the most studied tumor derived from TFH cells, various cytokines are secreted. Specifically, angiopoietin and vascular endothelial growth factor are secreted to induce proliferation, while interleukin (IL)‐5 is secreted to promote inflammation, including eosinophil proliferation.^1^ Accordingly, we assessed HEV proliferation and the number of eosinophils, which are representative infiltrative inflammatory cells. There was significantly higher HEV proliferation in ATLL with TFHP than in ATLL without TFHP. There was no significant between‐group difference in the eosinophil counts. Consistent with our findings, a previous study reported no difference in eosinophil infiltration between AITL and PTCL unspecified (currently known as PTCL, NOS).^14^ These histological features were generally the same as those of the previously reported study.^13^

Regarding FOXP3, the normal counterpart cell of ATLL is considered to be CD4 + CD25 + FOXP3 + T regulatory cells (Treg), and these cells are of a different lineage from TFH cells. While FOXP3 is considered a key molecule in Treg cells,^15^ previous studies have reported that ATLL do not always express FOXP3.^8^ Additionally, RHOA mutations, which are important for differentiation into T‐follicular helper cells, have been reported in approximately 15% of ATLL cases.^5^, ^6^ Therefore, it was hypothesized that part of ATLL might be TFH cells. If the expression of TFH markers and the expression of FOXP3 are inversely correlated, it suggests the possibility that a subset of ATLL is of TFH cell origin. However, we did not observe a relationship between the expression of TFH markers and FOXP3.

Regarding clinical presentation, cases of ATLL with TFHP showed increased CRP levels and a tendency to develop hypercalcemia. Regarding prognosis, there was no significant between‐group difference in OS. We observed no significant between‐group differences in other clinical parameters. Although ATLL with TFHP showed similar immunohistochemical characteristics, including TFH markers, as AITL, we observed no between‐group differences in the presence of hepatosplenomegaly, skin rash, and pleural effusion, which are the clinical characteristics of nTFHL‐AI. nTFHL‐AI involves immune activation and may be related to autoimmune disorders, including the presence of autoantibodies, hemolytic anemia, and hypergammaglobulinemia; however, we observed no significant between‐group difference.^16^ Contrastingly, we observed a significant between‐group difference in CRP levels. CRP is an acute‐phase protein produced in the liver, with its synthesis being induced by cytokines such as IL1β, IL6, and tumor necrosis factor (TNF). Lymphoma cells in AITL have been shown to produce TNFα and IL6.^14^, ^17^ Furthermore, lymphoma cells in ATLL with TFHP may produce these inflammatory cytokines; however, this was not investigated in this study since it deviated from the study objective. Although a previous study reported that the prognosis is worse with higher CRP levels, the prognosis of the TFHP group with high CRP levels was not significantly different from that without TFHP in our study.^18^ This may be due to the number of cases in this study not statistically sufficient. It is hoped that similar cases will be accumulated and re‐examined in a larger cohort.

There has been recent progress in the stratification of treatment for PTCL. Identified effective drugs include 5‐azacytidine and romidepsin, which have shown a higher overall response rate and CR rate in lymphoma with TFHP than without.^19^ Therefore, the treatment of ATLL should be stratified according to the presence of the TFHP.

This study had some limitations. First, there was no significant difference in OS, but this may be attributed to the limited sample size. Second, the chemotherapy regimens were not standardized between the ATLL cases with TFHP and those without TFHP: mSLG15 4 cases (18.2%) in TFHP vs. 14 cases (37.8%) in without TFHP, CHOP‐like regimen 9 cases (40.9%) vs. 10 cases (27.0%), VCAP‐AMP‐VECP 8 cases (36.4%) vs. 11 cases (29.7%), VP‐16 0 case (0%) vs. 2 cases (5.4%), and the former include 1 case (4.5%) with unknown chemotherapy details. In the future, we hope to accumulate a larger number of cases treated under standardized chemotherapy protocols.

## 5 CONCLUSION

Adult T‐cell leukemia/lymphoma may present with TFH markers, which does not preclude the diagnosis of ATLL but does not affect the prognosis. However, it may be important to stratify treatment interventions for ATLL according to the presentation of TFHP.

## AUTHOR CONTRIBUTIONS

**Reiji Muto:** Conceptualization (equal); data curation (equal); formal analysis (equal); investigation (equal); methodology (equal); project administration (equal); supervision (equal); validation (equal); visualization (equal); writing – original draft (equal). **Hiroaki Miyoshi:** Conceptualization (equal); investigation (equal); methodology (equal); resources (equal); supervision (equal); validation (equal); writing – review and editing (equal). **Kazutaka Nakashima:** Investigation (equal); methodology (equal). **Mai Takeuchi:** Methodology (equal); writing – review and editing (equal). **Makoto Hamasaki:** Writing – review and editing (equal). **Koichi Ohshima:** Writing – review and editing (equal).

## CONFLICT OF INTEREST STATEMENT

The authors have no conflict of interest.

## Data Availability

The data that support the findings of this study are available from the corresponding author upon reasonable request.
